# Delayed Facial Nerve Palsy after Vestibular Schwannoma Surgery in the COVID-19 Pandemic

**DOI:** 10.1055/s-0046-1819641

**Published:** 2026-04-30

**Authors:** Zdeněk Fík, Zuzana Balatková, Lenka Peterková, Karolína Hrubá, Kristýna Pospíšilová, Adéla Bubeníková, Eduard Zvěřina, Jan Betka, Vladimír Beneš, Aleš Vlasák

**Affiliations:** 1 Department of Otorhinolaryngology and Head and Neck Surgery, First Faculty of Medicine, Charles University, Motol University Hospital, Prague, Czech Republic; 2 Department of Neurosurgery, Second Faculty of Medicine, Charles University, Motol University Hospital, Prague, Czech Republic

**Keywords:** vestibular schwannoma, facial nerve, delayed palsy, COVID-19, SARS-CoV-2

## Abstract

**Introduction:**

Facial nerve palsy is the most common complication after vestibular schwannoma surgery. Its etiology is unknown and, in a small group of patients, it can manifest as delayed palsy.

**Objective:**

To focus on delayed palsy itself and its possible association with severe acute respiratory syndrome coronavirus 2 (SARS-CoV-2) infection.

**Methods:**

Only patients with primary surgery were included in the current study. Delayed palsy was defined as the new onset of facial nerve palsy that occurred more than 72 hours after surgery. The results were correlated with information from the database of the Institute of Health Information and Statistics of the Czech Republic.

**Results:**

From January 2017 to December 2022, a total of 194 patients were operated on for vestibular schwannoma, out of whom 143 met the inclusion criteria. Delayed facial nerve palsy occurred in 9 patients, more frequently in the coronavirus disease 2019 (COVID-19)-related years, with a ratio of 1:8 ( *p* = 0.0046) when comparing the periods of 2017 to 2019 and 2020 to 2022. There was a difference in the age of patients with delayed palsy (average: 44.0 years) and those without delayed palsy (average 49.9 years; *p* = 0.04). The age distribution in the individual years did not differ ( *p* = 0.38); however, the distribution of patients coincided with the overall appearance of SARS-CoV-2 infection in the population across different age groups ( *p* < 0.0001).

**Conclusion:**

Delayed facial nerve palsy is a rare entity after vestibular schwannoma surgery. The increase in its incidence during the COVID-19 pandemic is evident and corresponds to the general epidemiological situation with respect to age distribution.

## Introduction

Palsy of the seventh cranial nerve (nVII), also known as the *facial nerve* , is the most common complication after vestibular schwannoma (VS) surgery. In addition to the psychological impact, it severely affects both oral intake and eye lubrication, which can lead to long-term dreaded complications. 1 Generally, the risk of postoperative palsy increases together with tumor size; on the other hand, immediate postoperative malfunction is not a definitive result, and we are very likely to see an improvement in function. 2 However, the more severe the palsy, the less likely it is to return to normal function. 3

On the other hand, excellent facial nerve function after surgery does not necessarily mean an immutable state. In a small group of patients, delayed facial nerve palsy (DFNP) can be observed, in which the onset of palsy occurs later than 3 days after surgery. It is not rare that it affects patients who have primarily good/intact postoperative facial nerve function. 4

There are many theories about the etiology of delayed palsy; however, reactivation of herpetic viruses is the most suspected cause. 5

Treatment consists of a wide range of pharmaceuticals: anti-edematous, anti-inflammatory, and antiviral therapy together with physical therapy and, preferably, psychotherapy. 4

Fortunately, most patients experienced rapid improvement and returned to normal facial nerve function.

The severe acute respiratory syndrome coronavirus 2 (SARS-CoV-2) pandemic brought many new pathognomonic relations to hitherto known diseases due to the attack of the immunologically-naive population. However, in many cases, the relationship between the onset of some disease and infection/vaccination cannot be sufficiently proven. In the case of facial palsy, an association with infection or vaccination has been suspected. 6 7

We observed an increase in the incidence of delayed nVII palsy during the coronavirus disease (COVID-19) pandemic. The present article focuses on delayed palsy itself and its possible association with coronavirus infection.

## Methods

A retrospective analysis was performed to identify all patients who underwent VS surgical excision between January 2017 and December 2022 at the (name of department). The diagnosis of VS was based on the specific characteristics observed on magnetic resonance imaging (MRI), with confirmation by histopathological analysis.

Only patients who met the criteria of having normal preoperative facial nerve function, no history of radiation, and postoperatively intact anatomical facial nerves were included in the study. Furthermore, only patients after retrosigmoid suboccipital approach with gross-total resection were involved. The facial nerve outcomes were assessed using the House-Brackmann (HB) scale, measured from the time the patient fully regained consciousness after anesthesia.

Delayed facial nerve palsy was defined as a deterioration in function of one or more HB grades observed after more than 72 hours after surgery. The primary outcome measure was the HB grade at postoperative follow-up. Clinical data was collected, including patient age, sex, tumor size, facial nerve position, SARS-CoV-2 virus infection, and related vaccination. Patients without sufficient follow-up data were excluded from the study. A secondary analysis was performed, dividing DFNP patients into 2 groups: one for 2017 to 2019 and the other for the “COVID years” 2020 to 2022.

**Fig. 1:**
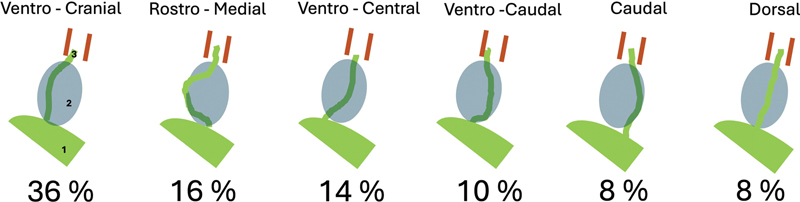
Different position of the facial nerve related to the tumor on the right side, sorted by frequency of occurrence. 1–brainstem, 2–tumor, 3–facial nerve.

The description of facial nerve position has been modified according to Bae et al. 8 and Sampath et al., 9 and it consists of six different locations of the nerve in relation to the tumor, which have been described previously 3 ( Fig. 1 ).

The results of the study were compared with data from the Institute of Health Information and Statistics of the Czech Republic ( https://onemocneni-aktualne.mzcr.cz/api/v2/covid-19 ). The following descriptive statistical methods were used for statistical analysis: mean, variance, and median categorical variables were described using mode and normalized entropy. The square test and the likelihood g-test were used to analyze categorical data from methods of inductive statistics. Both the Bravais-Pearson and the Spearman correlation coefficients were used to analyze the metric data. The F-test and the Bartlett test were used to verify the agreement of variances. The *t* -test, analysis of variance (ANOVA), and Mann-Whitney U test were used to statistically test the agreement of mean values and medians. To classify the age groups, we used the K-Means cluster analysis method. Statistical analysis was performed in Python 3.11 (Python Software Foundation) using the numpy, pandas, scipy.stats and pingouin, and scikit-learn libraries.

## Results

**Fig. 2:**
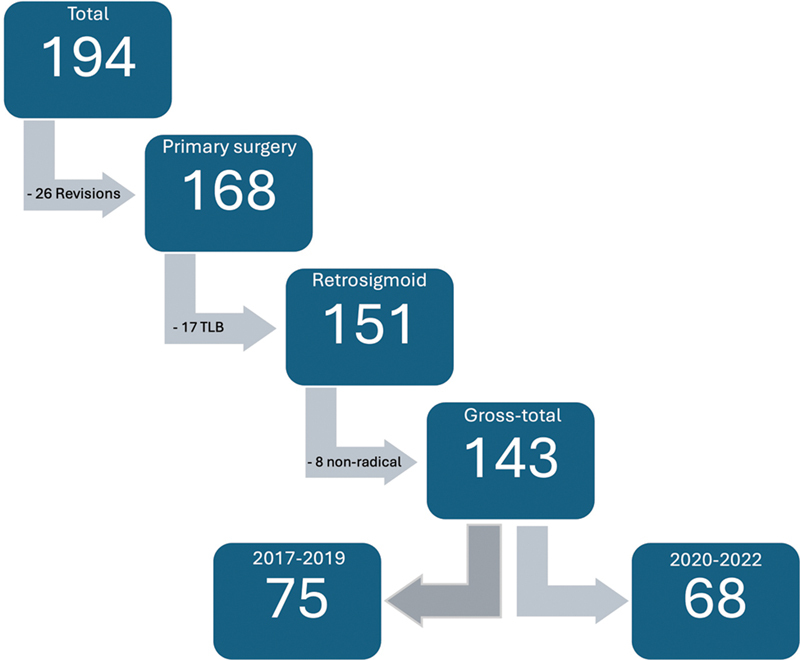
Flowchart – inclusion criteria; TLB – translabyrinthine.

From January 2017 to December 2022, 194 patients in total were operated on for VS. There were 14 patients who were operated on for recurrence or after failed stereoradiotherapy and were, therefore, excluded from the study. In 12 patients, the facial nerve has been disrupted. The retrosigmoid suboccipital approach (RS) was dominant in 151 patients, and the rest ( *n* = 17) were operated using the translabyrinthine approach (TLB). In 8 cases (5.6%), near-total or sub-total excision has been performed (all with the RS approach), as in the latter cases, a gross-total resection has been achieved. Thus, 143 patients were finally included in the study ( Fig. 2 ).

There were 73 women and 70 men, with an average age of 48.9 (12–74) years. Regarding the characteristics of the tumors, 69 were on the right side and 74 on the left. Larger tumors were more common, with 55 in the Koos 4 classification, 38 Koos 3, 43 Koos 2, and 7 Koos 1.

**Fig. 3:**
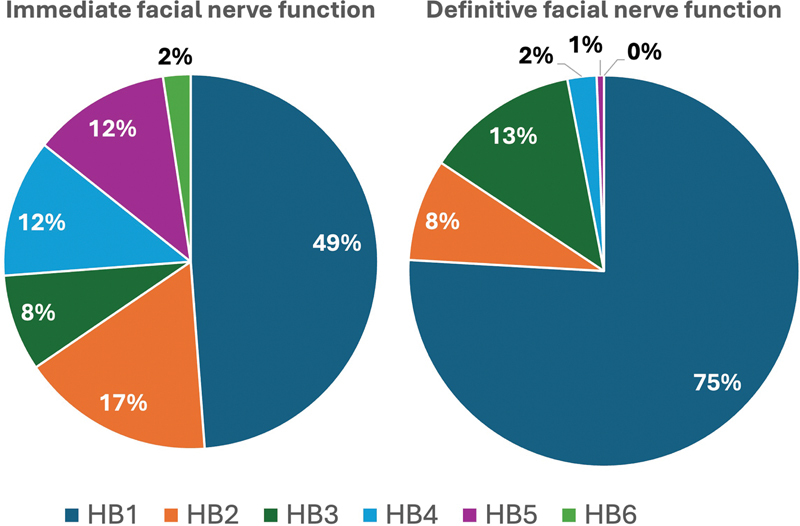
Immediate and definitive postoperative facial nerve function according to the House-Brackmann scale (HB1-HB6).

Immediate postoperative facial nerve function without size matching was good to normal (HB1–3) in 124 patients (74%). For the complete distribution, see Fig. 3 . Most of the patients showed improved HB degree, with definitive function HB1–2 in 96% ( Fig. 3 ). Immediate facial nerve palsy was correlated with tumor size, with average HB 3.2. in Koos-4 tumors, 2.3 in Koos-3 tumors, 1.7 in Koos-2 tumors, and 1.6 in Koos-1 tumors ( *p* = 4.4e-06).

The most common course of the facial nerve along the tumor was ventrocranial (36%), and the least common were the caudal and dorsal positions (8% each; Fig. 1 ). The dorsal position of the facial nerve was significantly correlated with the worst postoperative function ( *p* = 0.028).

**Fig. 4:**
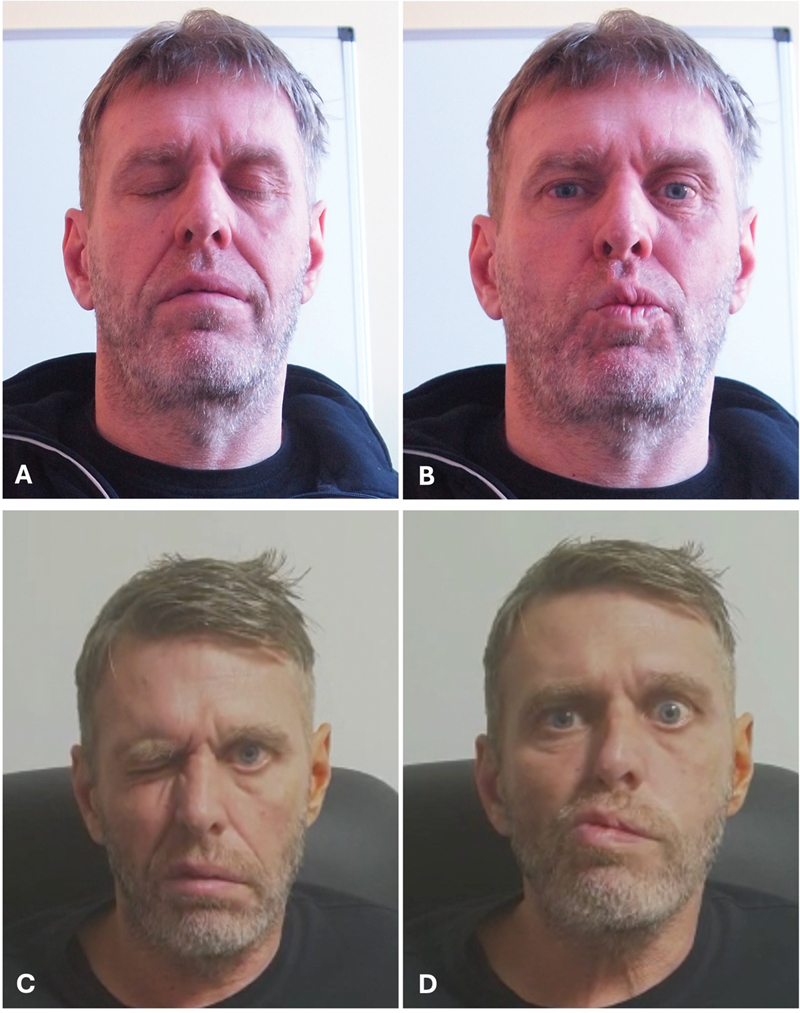
Patient with delayed palsy; ( **A,B** ) Immediate facial nerve function; ( **C,D** ) delayed facial nerve palsy. This patient improved to HB1 (not shown).

Delayed facial nerve palsy occurred in 9 patients ( Fig. 4 ), 1 case in 2017, 2 in 2021 and 6 in 2022. Immediate postoperative facial nerve function was normal (HB1) in 5 patients in the delayed palsy group. Three patients had HB2 and 1 had HB3. The onset of delayed palsy ranged from 4 to 14 days after surgery.

In 8 patients, facial nerve function recovered to HB1 (89%); in 1 case, it remained at HB4 16 months after surgery. For facial palsy dynamics, see Table 1 .

**Table 1:** Development of delayed facial nerve palsy in 9 patients (P1–P9)

	P1	P2	P3	P4	P5	P6	P7	P8	P9
Immediate nVII function	HB 1	HB 2	HB 2	HB 1	HB 2	HB 1	HB 3	HB 1	HB 1
Delayed palsy in nVII function	HB 5	HB 3	HB 4	HB 2	HB 6	HB 5	HB 6	HB 3	HB 5
Definitive nVII function	HB 1	HB 1	HB 1	HB 1	HB 1	HB 1	HB 4	HB 1	HB 1

**Abbreviation** : HB, House-Brackmann scale; nVII, seventh cranial nerve.

None of the patients with delayed palsy reported symptoms of COVID-19 and only 2 of them underwent laboratory-proven SARS-CoV-2 infection. In one case, more than 1 year prior to surgery. In the second case, the positive test was performed in a non-symptomatic patient who was scheduled to undergo surgery, which had to be postponed for 2 weeks.

From January 2020, 16 patients (23.5%) underwent laboratory-proven SARS-CoV-2 infection, with an average time of 263 days before surgery (15–489 days). During the same period, 30 patients underwent a complete vaccination scheme before surgery (44.1%), with an average time of 273 days (15–518 days). Comirnaty (Pfizer, BioNTech) has been administered in 26 patients, Spikevax (Moderna) in 3, and Janssen (Johnson & Johnson) in 1. Of the group of patients who experienced infection, 12 were also vaccinated throughout the scheme (75%).

Delayed facial nerve palsy occurred in 9 patients and more frequently in years related to COVID-19, with a ratio of 1:8 ( *p* = 0.0046; power = 0.56) when comparing the periods of 2017 to 2019 and 2020 to 2022.

No relationship has been observed between delayed palsy and sex or tumor size.

Interestingly, there was a difference in the age of patients with delayed palsy (average: 44.0 years) and those without it (average 49.9 years; *p* = 0.04; power = 0.72). The age distribution in individual years did not differ ( *p* = 0.38; power = 0.38). Patients with delayed palsy were generally younger. We were unable to confirm a correlation with age when focusing only on patients with proven SARS-CoV-2 infection ( *p* = 0.29; power = 0.68) or completed vaccination ( *p* = 0.294; power = 0.42). When focusing on data from the Czech Republic Institute of Health Information and Statistics in the study period, the incidence of infection was higher in the younger part of the population. Patients with ages similar to or below 40 years were at a significantly higher risk of infection compared with patients in older groups ( *p* -value < 0.0001; power > 0.99).

## Discussion

Delayed facial nerve palsy following VS resection is an unpleasant complication; typically, patients observe an increase in facial weakness in real time after an initially normal postoperative period. The definition of DFNP varies in the literature. The most common time of onset is suggested to be 72 to 120 hours after surgery, 4 10 with a worsening of the HB scale of at least one degree. 11 However, in certain publications, deterioration after 24 hours is still considered DFNP, whereas in others, the diagnosis of DFNP requires a decrease of two or more grades in the HB classification. 4 Some studies consider each deterioration after initial postoperative facial nerve evaluation as DFNP. 11 Regarding another aspect, 2 types of delayed palsy can be described: early onset (< 48 hours) and late onset (≥ 48 hours), with the former typically associated with a poorer prognosis for complete recovery. 12 13 Furthermore, if DFNP affects patients who present with immediate (even mild) postoperative palsy, the prognosis is worse than in those with normal facial nerve function in the immediate postoperative period. 10

Based on our experience, the most accurate interval between surgery and the onset of DFNP is 72 hours, excluding patients with direct injury caused by surgical trauma leading to axonal degeneration. This degeneration requires a longer recovery period, and, therefore, affected patients usually do not follow one of the typical patterns of this disorder—rapid recovery between 4 and 6 weeks. 12 From this point of view, the reactivation of the herpetic virus seems to be the most suspected element that leads to demyelination. 12 Remyelination takes a shorter time to restore; thus, a shorter period of transient palsy can be expected. In addition, herpetic infection has been proven in some serological studies. 5 Furthermore, a study described differences in facial nerve enhancement in the labyrinthine segment when comparing the delayed and non-delayed palsy groups. This higher enhancement is comparable to findings in Bell's palsy patients. 14 In addition, DFNP is probably less likely to be seen in patients after translabyrinthine or subtemporal approach if the labyrinthine segment (of the facial nerve) has been decompressed. 15 16 The retrosigmoid approach is considered to have a higher risk of DFNP development in some studies, 4 but this statement is inconsistent with the findings of MacDonald et al., who compared the retrosigmoid and translabyrinthine approach and found that the latter was more often associated with DFNP. 17 Lalwani et al. and Grant et al. found no correlation with the approach used. 11 18 We did not consider this in our study, as there was a relatively low number of TLB surgeries. On the other hand, a study suspected that postoperative hematoma at the cerebello-pontine angle is associated with a higher risk of developing DFNP. 19

Delayed facial nerve palsy has been reported to occur in 4.8 to 41% of patients after VS surgery; however, the most common reported range lies between 10 and 25%, 12 13 17 20 which is quite a high number, compared with the one found in our study—5.4% overall and 1% during the prepandemic years. In the years of the COVID-19 pandemic, 2020 to 2022, the incidence of DFNP was 10% in our study. The explanation probably lies in the definition discussed above, as many patients in the aforementioned studies experienced palsy in the first 3 days after surgery.

The prognosis of DFNP is generally very good; the recovery range to grade HB1–2 varies between 71% and 100%, which is comparable to our study (89%). 4 10 17 21

Despite the statements above, the treatment of DFNP remains controversial. Some studies recommend immediate antiviral therapy, while others only advocate for anti-edematous therapy (corticosteroids) or a non-active approach with spontaneous recovery. 5 11 22 Carlstrom et al. did not find any differences in the treatment strategies mentioned above. 4

The SARS-CoV-2 pandemic has brought several new etiopathogenetic entities, with the involvement of the peripheral nervous system being one of them; however, the mechanism remains unclear to date. 23

The involvement of the nervous system during a SARS-CoV-2 infection is estimated at up to 36.4% of cases. 24 It can be divided into palsies associated with infection or vaccination. The most commonly affected is the olfactory nerve with disturbances in the sense of smell. 25 It is debatable whether taste disorders come hand in hand with the latter due to retronasal taste perception or is a separate entity—the affection of the gustatory cells themselves. 26 Several peripheral nerve palsies have been reported. As the olfactory sensory cells are part of the central nervous system, the most common peripheral nerve affected by COVID-19 infection is the facial nerve. Ocular cranial nerves (the third [nIII], fourth [nIV], and sixth [nVI] nerves) have also been frequently reported. 27 28 29 30 Besides other cranial nerves, it has been reported that the twelfth nerve (nXII) is associated with complications related to COVID-19. 31

As Khatami et al. further detailed peripheral nervous system complications after COVID-19 vaccination, including facial nerve palsy, as part of broader inflammatory neuropathic conditions, the etiology of cranial nerve palsies in association with COVID-19 infection is quite unclear. 32 The studies that address this issue are, almost without exception, based on case reports. Moreover, all epidemiological studies on Bell's palsy incidence increase during the pandemic are lacking direct proof of COVID-19 infection in patients with facial nerve palsy. 6 33 34 Direct neural damage caused by the SARS-CoV-2 virus is hypothesized, as well as indirect affection, based on immunological or autoimmune reactions (e.g., herpetic virus reactivation). 29 30 Other case reports showed suspicious vessel involvement related to nerve ischemia with respect to the ability of the SARS-CoV-2 virus to increase the pro-coagulatory state. Furthermore, central nervous system vasculitis could also be associated with isolated cranial nerve palsy. 27 Finally, mechanistic causes must be considered in intensive care units (ICU) patients when there is a need for positioning, which increases the risk of nerve compression (e.g., ulnar nerve). 23

The incidence of idiopathic facial nerve palsy increased during the COVID-19 pandemic. According to 1 study, it shifted from 1.3% in 2019 to 3.5% in 2020. 34 A similar trend has been observed in the Italian and Chinese populations, 6 33 and it has been proven that the SARS-CoV-2 virus is the direct affecting factor. 35 36 37 Kwak et al. found a significant increase in Bell's palsy cases during the pandemic, particularly among elderly diabetic patients, with decreased full recovery rates and increased recurrence compared with the pre-pandemic era. 38 A meta-analysis by Mirza et al. reported a significantly increased incidence of facial nerve palsy during the COVID-19 pandemic compared with the pre-pandemic period. 39 On the other hand, both Lassaletta et al. and El-Deeb et al. observed no overall increase in Bell's palsy incidence in association with COVID-19 infection; however, suggesting possible influence of the clinical course of the palsy. 40 41

The responsible mechanism is still unknown. Two main pathogenetic mechanisms are discussed in the literature. First, direct neurotropic damage to the nerve by coronavirus has been identified, with its increased affinity for the angiotensin-converting enzyme 2 receptors present on peripheral nerves. 42 The gateway probably lies in the presence of sensorimotor facial nerve fibers in the nasal mucosa. 43 Direct damage can then occur, for example, as the result of coronavirus' OC43 protein axonal transfer. 44 Such mechanism has been supported by Wang et al., who depicted similarities with other neurotropic viruses, such as HSV-1 and VZV. 45

The second mechanism proposed is an inappropriate immunological response, which can cause cytokine release syndrome, leading to neuronal damage. 46 Furthermore, cytokines can induce microthrombosis in the nervous system blood supply, which can cause or promote facial nerve damage. 47

However, other underlying causes that increase the sensitivity to infection must also be taken into account (tumor, compression). 48

In addition to infection, vaccination has been considered another etiopathogenetic factor of facial nerve palsy associated with SARS-CoV-2 infection. 49 50 Yoon et al. reported an increased risk of facial palsy post vaccination across multiple vaccine types, based on a nationwide self-controlled case series in South Korea. 51 Hamid et al. described imaging- confirmed cases of acute facial palsy occurring shortly after mRNA vaccination, reinforcing the plausibility of a post-vaccination inflammatory neuropathy. 52 On the contrary, Mirza et al. did not find a significant association with vaccination alone; nevertheless, in a separate review, they analyzed 52 individual cases of post-vaccination facial nerve palsy and found that most occurred within one week of receiving mRNA vaccines. These cases were typically mild and unilateral, although the full recovery rate (55%) was notably lower than the pre-pandemic standard of 83%. 39 53 However, according to other studies, the association of facial palsy and vaccination is probably comparable or even lower in incidence compared with influenza vaccines. 54

Direct neural damage is probably the mechanism responsible for isolated cranial nerve palsies, as the indirect effect may lead to a more complex neurological disorder, such as Guillain-Barré syndrome (GBS). 55 56 Navamarian et al. described a higher proportion of patients with nVII palsy together with GBS (up to 42%). Furthermore, this combination has been associated with the worst recovery prognosis. On the other hand, isolated nVII palsy occurred in a shorter period compared with classic Bell's palsy and was more often bilateral. Finally, up to 11% of those who were tested positive for COVID-19 had facial nerve palsy as the only symptom. 36 A better prognosis of COVID-19 affection over Bell's palsy is also suggested in a paper by Egilmez et al. 57

Isolated case reports describe the combination of perimyocarditis together with nVII palsy. Interestingly, the palsy came as a late-onset symptom after the cardiac manifestation (> 10 days). 58 59

There are some limitations of the current study. Due to its retrospective design, it was not possible to reach a higher proportion of SARS-CoV-2-tested patients before surgery. However, in other population studies on facial nerve palsy during pandemics, a direct association with COVID-19 infection is also lacking. Furthermore, the low number of DFNP in our study corresponds to our long-term experience and is particularly influenced by our strict adherence to the 72 hours interval between surgery and the onset of palsy. The division into 2 groups (2017–2019 and 2020–2022) offers a comparable number of patients, but the clearance of infection in the population in 2020 was indeed not comparable to that of 2022. Nevertheless, the dynamics of DFNP incidence in particular years followed the overall spread of the infection in the

## Conclusion

Severe acute respiratory syndrome coronavirus 2 infection has been shown to be associated with facial nerve palsy. To date, no study has focused on its association with facial nerve palsy after surgical procedures, such as vestibular schwannoma surgery. We observed a higher proportion of delayed palsies after VS surgery in the COVID-related years, where age was the only connecting element, as younger patients were at higher risk of delayed palsy. We did not find any direct explanation for this statement; however, the distribution of patients coincided with the general appearance of SARS-CoV-2 infection in the population divided by age.
